# Exploring the Potential of Artificial Intelligence (AI) and Augmented Reality (AR) in Neurological Disease Management: A Narrative Review

**DOI:** 10.7759/cureus.86614

**Published:** 2025-06-23

**Authors:** Jennifer Ifeoma Anene, Abhimanyu Sharma, Arunveer Sharma, Iram Fatima, Neeraj Kancherla, Sarath Chandra Ponnada, Sai Krishna Ravuru, Christina Thymalil, Soura Rajeshwara, Anchit Chauhan, Shreya Muralidharan, Anirudh FNU, Ballu R

**Affiliations:** 1 Internal Medicine, Ivano-Frankivsk National Medical University, Ivano-Frankivsk, UKR; 2 Internal Medicine, SGRD (Sri Guru Ram Das) Institute of Medical Sciences and Research, Amritsar, IND; 3 Nursing, Toronto Metropolitan University, Toronto, CAN; 4 Internal Medicine, Holy Name Medical Centre, Teaneck, USA; 5 Internal Medicine, Philadelphia College of Osteopathic Medicine, Philadelphia, USA; 6 General Medicine, Great Eastern Medical School and Hospital, Srikakulam, IND; 7 Medicine, Great Eastern Medical School and Hospital, Srikakulam, IND; 8 Internal Medicine, Dr. Kiran C. Patel College of Osteopathic Medicine Nova Southeastern University (NSU-KPCOM), Fort Lauderdale, USA; 9 Psychiatry, Shimoga Institute of Medical Sciences, Shivamogga, IND; 10 Internal Medicine, Maulana Azad Medical College, New Delhi, IND; 11 Internal Medicine, Vydehi Institute of Medical Sciences and Research Centre, Bangalore, IND; 12 Internal Medicine, All India Institute of Medical Sciences, Bathinda, Bathinda, IND; 13 Neurology, University of Florida, Gainesville, USA

**Keywords:** ai ethics, digital twins, neurodegenerative disorders, neurological diseases, neurorehabilitation

## Abstract

Recent advancements in artificial intelligence (AI) and augmented reality (AR) hold significant promise for revolutionizing the management of neurological diseases. These technologies enable enhanced diagnostic accuracy, personalized treatment plans, and innovative rehabilitation strategies. AI-driven systems can analyze complex neuroimaging data, track disease progression, and assist in neuroprosthetic interventions, while AR improves surgical precision and supports interactive, immersive training for healthcare professionals. This narrative review explores the current and potential applications of AI and AR in neurology, focusing on their impact on diagnosing and treating neurodegenerative disorders, stroke, and other neurological conditions. We also address the challenges of data security, bias in AI models, and the high cost of AR devices, along with the ethical implications of integrating these technologies into clinical practice. The review concludes with a discussion of future directions, emphasizing the need for larger, long-term studies, interdisciplinary collaborations, and policy frameworks to ensure the safe, equitable, and effective implementation of AI and AR in neurological care.

## Introduction and background

In recent years, the integration of augmented reality (AR) and artificial intelligence (AI) has transformed the healthcare landscape, particularly in disease diagnosis, understanding, and management. AR enhances real-world perception by overlaying computer-generated information, modifying visual, auditory, and sensory input, while AI enables machines to perform tasks that traditionally require human intelligence [1,2]. Together, these technologies are shaping a new paradigm in clinical practice.

Over the past four decades, AR has demonstrated utility in medical education and procedural training. AI, meanwhile, has made strides in clinical decision-making through data-driven pattern recognition and prediction, notably accelerated by deep learning (DL) models. These systems can process complex, large-scale datasets to uncover clinically relevant insights that were previously inaccessible. Landmark initiatives, such as IBM Watson (IBM Corp., Armonk, NY) and Google DeepMind (Google, Deepmind Holdings Limited, London, UK), have illustrated the potential of AI in managing diabetes, interpreting medical imaging, and tailoring cancer therapies [3].

Despite this potential, the adoption of AR and AI in healthcare, especially in neurology, remains limited [4]. Neurological diseases encompass a broad spectrum of conditions affecting the brain, spinal cord, and peripheral nerves, with millions impacted globally each year [5]. Given the complexity of neurologic data, spanning history, imaging, labs, and genomics, AI offers a promising tool for advancing precision medicine in this domain. Similarly, AR provides neurosurgeons with real-time, spatially accurate visualizations that enhance operative precision and safety [6]. This review aims to explore the current applications and future potential of AR and AI technologies in the diagnosis, management, and education surrounding neurological diseases and neurosurgical practice. By synthesizing available evidence, we aim to inform clinicians, educators, and researchers about emerging innovations and guide future integration of these technologies into neurologic care.

## Review

This narrative review investigates the current and potential applications of AI and AR in the management of neurological diseases. A comprehensive literature search was conducted across PubMed, Scopus, IEEE Xplore, and Web of Science databases, covering publications from May 2015 to May 2025. The search strategy included a combination of Medical Subject Headings (MeSH) and keywords such as “Artificial Intelligence,” “Augmented Reality,” “Neurological Diseases,” “Neurodegenerative Disorders,” “Alzheimer’s,” “Parkinson’s,” “Stroke Rehabilitation,” and “Cognitive Impairment.” Boolean operators (AND, OR) were used to refine and focus results on studies intersecting AI or AR with the diagnosis, treatment, or rehabilitation of neurological conditions. The search was limited to peer-reviewed English-language articles. Studies were included if they examined the effectiveness or clinical application of AI or AR in neurological practice and were excluded if they did not pertain to these technologies or focused on non-neurological domains. Screening and selection of studies were conducted independently by two reviewers, with a third reviewer resolving any disagreements. This process was blinded and performed in duplicate to minimize bias. Quality assessment tools were applied based on study design: the Cochrane Risk of Bias Tool for randomized controlled trials (RCTs), the Newcastle-Ottawa Scale for observational studies, and the Technology Readiness Index (TRI) for evaluating the maturity and clinical applicability of technological interventions. Author roles were defined as follows: one reviewer (Author A) led the search strategy and database extraction, another (Author B) refined MeSH terms and Boolean logic, and a third (Author C) oversaw quality assessment and resolved conflicts during screening. This methodological approach ensures a structured and reproducible synthesis of current literature, offering meaningful insights into the evolving role and clinical impact of AI and AR in neurological disease management.

Current applications of AI

Advancements in artificial intelligence, particularly machine learning and deep learning techniques, have shown promising results in improving the diagnostic accuracy and evaluation of these conditions [7]. AI-driven systems analyze either neuroimaging data or patient responses during rehabilitation and then make real-time adjustments to therapies to tailor interventions to meet the specific needs of individuals [8].

Natural language processing (NLP) algorithms and automated lesion detection networks (described in the next section) may augment efficiency in triaging imaging studies of patients with acute, time-sensitive presentations such as stroke. Current AI tools on the market can send processed imaging data, such as CT perfusion maps, via text messages to stroke physicians and neurointerventional radiologists in real-time [9]. The progression and diagnosis of multiple sclerosis (MS) are dependent on neuroimaging. In this context, AI holds significant promise for accurately quantifying and tracking lesions in individual patients. A variety of research AI models (including NNs, k-Nearest Neighbors, and support vector machines (SVM)) exist for detecting MS lesions with high specificity and sensitivity [10].

Digital twin technology is an emerging concept in neuroscience that holds significant promise for advancing personalized medicine and understanding brain function. A digital twin refers to a highly detailed virtual model of a physical object, system, or process that is continuously updated using real-time data. In neuroscience, digital twins can replicate anatomical structures or simulate dynamic physiological processes such as neural activity patterns. These models allow researchers and clinicians to better understand individual brain function, predict the progression of neurological diseases, evaluate treatment responses, and design tailored interventions. Given the brain's complexity and the limitations of current diagnostic tools, digital twins offer a powerful method for integrating multimodal data, including neuroimaging, electrophysiology, and clinical history, to improve precision in clinical decision-making [11].

Similarly, brain-computer interfaces (BCIs) are technologies that establish a direct communication link between the brain and external devices. By interpreting neural signals, AI-powered BCIs enable individuals with severe motor impairments, such as those with locked-in syndrome, to communicate, control wheelchairs, robotic limbs, or type on computers, thereby enhancing autonomy and quality of life [12]. These innovations are transforming neurorehabilitation and neuroprosthetics, especially when combined with digital twin modeling to optimize BCI design and functionality.

AI is making neurorehabilitation even more accessible by becoming part of telerehabilitation platforms. This advance enables constant monitoring and personalized treatment, even for patients residing in remote locations. AI-powered systems may provide instant feedback during therapy sessions, that is, by changing exercises and guidance that do not require a therapist to be on-site [13].

Current applications of AR in medicine

Overview of AR and Related Technologies

AR overlays digital content onto the physical world using devices like smartphones or headsets. It is part of extended reality (XR), which also includes virtual reality (VR), fully immersive environments, and mixed reality (MR), which blends digital and real-world elements. Together, XR technologies are advancing healthcare through enhanced visualization, education, and patient care.

Educational Applications

AR has proven effective in training medical, dental, and nursing students by offering interactive simulations [14-16]. In hospitals, it assists with procedural training in CPR, vascular access, intubation, and more [17,18].

Pediatric and Geriatric Use

AR reduces anxiety and pain in pediatric patients during ICU stays and preoperative education. For instance, Tait et al. found children and parents preferred AR-based materials over printed guides [19,20].

Rehabilitation

Gamified AR tools improve engagement in rehabilitation, including gait training for Parkinson’s patients [21,22]. Post-stroke and orthopedic rehab show improved outcomes, self-esteem, and patient-therapist interaction [21,23]. AR also supports post-amputation recovery and phantom limb pain management [24,25].

Surgical Applications

AR and VR support surgical training and intraoperative navigation across specialties, including neurosurgery, orthopedics, and interventional radiology. While technical limitations exist, these tools improve precision and postoperative outcomes [26-43].

Comparative analysis of AI vs. AR

AI and AR each offer unique advantages in neurological care. AI excels at processing large, complex datasets, enabling real-time analysis of imaging data, such as CT and MRI scans, and supporting clinical decision-making through predictive modeling. AR, on the other hand, enhances visualization by overlaying digital content onto the physical environment, providing intuitive, spatially contextual information that improves user interaction and comprehension. However, each technology presents distinct challenges: AI requires high-quality, annotated datasets and poses concerns related to interpretability and algorithmic bias, while AR is limited by hardware accuracy, restricted fields of view, and challenges in integrating into existing clinical workflows.

AI's ability to process complex neurological data, such as CT or MRI scans, in real-time provides the foundation for AR applications in surgical settings [44]. For instance, during neurosurgical procedures, AI algorithms can delineate tumor margins with high accuracy, which AR visually overlays onto the surgeon's field of view [45]. This synergy reduces risks and enhances precision, especially in intricate areas like the brainstem.

Neurological rehabilitation has benefited immensely from AI and AR collaboration. AI's analytics provide personalized insights into a patient’s progress, while AR platforms create immersive, interactive exercises tailored to specific neurological deficits, such as motor skill recovery in stroke survivors. For example, an AI-enhanced AR system can adapt a rehabilitation task in real-time based on patient performance, making therapy more engaging and effective [46]. AI’s predictive modeling capabilities complement AR’s visual storytelling.

In epilepsy management, the integration of AI and AR offers significant clinical benefits. AI algorithms can analyze continuous EEG or neuroimaging data to predict seizure patterns, allowing clinicians to anticipate episodes and plan interventions proactively [47]. During surgical procedures, AR enhances this by providing real-time, spatially accurate visualizations of seizure foci, superimposed onto the surgeon’s field of view [48]. This integration improves surgical planning and intraoperative navigation, enabling more precise localization and resection of epileptogenic zones. As a result, the combined use of AI and AR reduces the risk of incomplete excision or collateral damage to surrounding brain tissue, thereby enhancing surgical precision, minimizing complications, and improving long-term seizure control and neurological outcomes.

AR simulations have revolutionized training for neurological procedures, providing lifelike virtual environments [49]. When paired with AI, these simulations become more interactive, with adaptive scenarios that adjust to the learner’s actions. This dynamic learning model enhances skills acquisition and decision-making for medical professionals.

Challenges and ethical considerations

Despite their transformative potential, the integration of AI and AR in neurological care introduces several critical challenges and ethical considerations. AI systems depend on large volumes of sensitive data, such as brain imaging, EEG signals, and genetic profiles, which raises significant concerns about data security and privacy [50]. When paired with AR, which visualizes patient data in real time during clinical procedures, the risk of unauthorized access increases, particularly if the data streams are not adequately encrypted. Ensuring compliance with existing frameworks like the General Data Protection Regulation (GDPR) and the Health Insurance Portability and Accountability Act (HIPAA) is vital, yet these regulations were not originally designed for immersive technologies and may lack the specificity needed for AR and AI applications in healthcare settings. Regulatory updates tailored to intelligent and immersive tools are urgently needed to address issues such as real-time data visualization, AI decision accountability, and device interoperability.

Bias in AI algorithms remains a substantial concern. When models are trained on non-representative datasets, they may perpetuate or even exacerbate disparities in neurological care such as underdiagnosing stroke symptoms in women or minority populations [51]. Similarly, the high cost of AR hardware can restrict access to cutting-edge interventions in low-resource settings, widening healthcare inequities [52].

Concerns have also been raised about over-reliance on intelligent systems. For instance, studies have documented that clinicians may defer excessively to AI-generated outputs, especially under cognitive load, leading to automation bias and reduced diagnostic vigilance [53]. In neurosurgery, a misplaced AR overlay or incorrect AI-generated tumor margin could result in surgical errors with life-altering consequences. A review by Obermeyer et al. (2019) highlighted that unchecked dependence on opaque AI systems could compromise clinical judgment, particularly when algorithms lack transparency or interpretability [54]. These concerns underscore the need to maintain clinician oversight and encourage AI-AR use as augmentative tools, not replacements, for expert decision-making.

To ensure safe clinical integration, AI and AR systems must undergo rigorous validation in real-world environments. Regulatory bodies like the FDA and EMA are beginning to explore adaptive oversight frameworks, but current pathways remain fragmented and may lag behind the pace of innovation [55]. Ethical implementation also demands transparency, robust informed consent processes, and clear communication with patients about the capabilities and limitations of AI-AR tools [56]. Establishing protocols for error reporting, system accountability, and algorithm retraining will be essential to building trust and ensuring equitable care.

Future directions

The integration of AI and AR into neurological care offers immense promise, but critical research gaps remain. Many current studies have small sample sizes, short follow-ups, or lack diversity in patient demographics. To validate efficacy, safety, and generalizability, larger and longer-term studies across diverse populations are essential. For example, while AR-assisted neurosurgical tools have improved surgical precision, it remains unclear whether these benefits translate into long-term survival or quality-of-life improvements. Similarly, AI-based rehabilitation for stroke and Parkinson’s disease shows early promise, but long-term outcomes remain underexplored.

Brain-computer interfaces (BCIs) and AI-AR neuroprosthetics, capable of decoding and transmitting motor signals for limb control, are also gaining traction [57-59]. In the longer term, more complex technologies are expected to revolutionize personalized and predictive neurological care. Digital twins, virtual simulations of a patient’s neural system, could model disease progression in Alzheimer’s, multiple sclerosis, or spinal conditions to forecast outcomes and plan treatments [60,61]. AI-enhanced neuromodulation methods, such as adaptive deep brain stimulation (DBS) or transcranial magnetic stimulation (TMS), may tailor protocols to individual neural patterns, while AR could visualize effects in real time [62,63].

In the near future, we can expect scalable innovations that are already showing feasibility in clinical or home settings. Wearable AI-powered devices are emerging to detect subtle motor or behavioral changes in conditions like Parkinson’s and Huntington’s diseases [64-66]. These tools enable earlier intervention and personalized monitoring. Similarly, remote rehabilitation using AR environments with motion capture, guided by adaptive AI algorithms, allows stroke or spinal cord injury patients to progress at home without constant clinician supervision [67]. Integration with extended reality (XR), including AR, VR, and MR, is already being piloted for immersive neurological training in stroke or traumatic brain injury management [68,69]. These systems can provide real-time feedback and augment motor recovery in patients with paralysis. Drug discovery may also shift dramatically with AI models predicting drug-target interactions, and AR environments simulating neurological responses prior to clinical trials, reducing risk and cost [70].

Additionally, AI has the potential to enhance magnetic resonance spectroscopy and neuroimaging by identifying novel biomarkers from genomic, proteomic, and imaging data-especially for diseases like ALS and Alzheimer’s [71]. Emotional and cognitive assessments through multimodal AI analyses of EEG, facial expressions, and speech are under development for disorders like post-traumatic stress disorder (PTSD), attention deficit hyperactivity disorder (ADHD), and dementia [72,73]. Evolving imaging methods, such as functional MRI and diffusion tensor imaging, are also benefiting from AI’s ability to map activity and white matter integrity more accurately in conditions like traumatic brain injury and autism [74].

The integration of AI and AR into neurology is rapidly transforming patient care; however, ensuring their safe, equitable, and effective use requires addressing several key policy and regulatory challenges [75]. Protecting data privacy and security is essential, especially when dealing with sensitive information like brain imaging or genetic data. Regulatory frameworks must evolve from existing standards, such as GDPR and HIPAA, focusing on secure data transmission and storage [76]. Measures like mandating end-to-end encryption and blockchain-based systems could help safeguard patient information from unauthorized access and increase transparency in how data is handled [77]. To ensure that these technologies serve all populations equitably, policymakers must address biases in AI models. To address this, diverse and inclusive datasets must be used to train AI systems with apt periodic reviews. Similarly, accessibility challenges posed by the high costs of AR devices can be tackled if governments could promote public-private partnerships to subsidize AR technologies for underprivileged communities while encouraging manufacturers to develop affordable, universally accessible systems [78].

Establishing trust in AI-AR systems requires validation and monitoring. Regulatory agencies like the FDA and EMA must design flexible validation protocols that account for real-world variable conditions [79]. Ongoing post-market surveillance is crucial to detect and correct errors like inaccuracies in AI predictions [75]. In parallel, policymakers should prioritize ethical considerations surrounding the use of these technologies [80]. For example, guidelines must ensure that AI-AR tools remain assistive rather than replacing clinical expertise, particularly in high-stakes scenarios like neurosurgery. Awareness campaigns for healthcare providers about the limitations of AI-AR systems can prevent over-reliance on these technologies and promote balanced decision-making [81]. Ethical standards, as discussed in the prior section, should also keep up with advancements in technology: using AR-based informed consent systems and maintaining transparency about the use of these systems [82]. Further International collaboration among regulators could lead to global guidelines on critical issues like data ownership and algorithmic accountability, promoting the responsible use of these technologies in neurology. The future of AI and AR in neurology depends on policies by regulatory bodies that ensure data security, reduce biases, improve accessibility, and uphold ethical standards, which will increase trust in the adoption of these advancements.

## Conclusions

This narrative review highlights the transformative role of artificial intelligence (AI) and augmented reality (AR) in neurology, emphasizing their potential to enhance diagnostic accuracy, personalize treatments, and improve surgical precision. AI tools, such as brain-computer interfaces and telerehabilitation platforms, are expanding patient autonomy and access to care, while AR enables real-time brain visualizations that support complex neurosurgical procedures. When integrated, AI and AR create synergistic systems that optimize neurological care delivery. However, challenges, including high implementation costs, data security concerns, and disparities in access, must be addressed to realize their full potential. Future advancements in computational neuroscience, neuroprosthetics, and dynamic brain imaging hold immense promise. To ensure equitable, effective, and patient-centered integration of these technologies, we call on researchers to drive innovation grounded in clinical relevance, clinicians to advocate for responsible adoption in practice, regulators to establish clear standards for safety and privacy, and policymakers to support infrastructure and training initiatives. Only through coordinated, interdisciplinary action can AI and AR fulfill their potential to transform neurological care.
